# Surveillance for sickle cell disease, United Republic of Tanzania

**DOI:** 10.2471/BLT.20.253583

**Published:** 2020-09-28

**Authors:** Emmanuela E Ambrose, Luke R Smart, Mwesige Charles, Arielle G Hernandez, Teresa Latham, Adolfine Hokororo, Medard Beyanga, Thad A Howard, Erasmus Kamugisha, Kathryn E McElhinney, Erius Tebuka, Russell E Ware

**Affiliations:** a Department of Paediatrics and Child Health, Catholic University of Health and Allied Sciences, Mwanza, United Republic of Tanzania.; b Division of Hematology, Department of Pediatrics, University of Cincinnati College of Medicine, Cincinnati Children’s Hospital, 3333 Burnet Avenue, MLC 7015 Cincinnati, Ohio 45229, United States of America.; c Department of Laboratory Services, Bugando Medical Centre, Mwanza, United Republic of Tanzania.; d Department of Biochemistry and Molecular Biology, Catholic University of Health and Allied Sciences, Mwanza, United Republic of Tanzania.; e Department of Pathology, Catholic University of Health and Allied Sciences, Mwanza, United Republic of Tanzania.

## Abstract

**Objective:**

To determine the regional- and district-level newborn prevalence of sickle cell trait and disease, and the prevalence of haemoglobin variants and genetic modifiers of sickle cell disease, in the nine regions of north-western United Republic of Tanzania.

**Methods:**

We repurposed dried blood spot samples from children (aged 0–24 months) born to mothers living with human immunodeficiency virus (HIV), collected as part of the HIV Early Infant Diagnosis programme, for sickle cell diagnosis. We performed isoelectric focusing to determine whether samples had normal haemoglobin, sickle cell trait, sickle cell disease or a rare haemoglobin variant. We shipped samples diagnosed as disease or variant to Cincinnati Children’s Hospital in the United States of America for deoxyribonucleic-acid-based analyses to determine the prevalence of α-thalassaemia, glucose-6-phosphate dehydrogenase (G6PD) deficiency or fetal haemoglobin genetic modifiers.

**Findings:**

We analysed a total of 17 200 specimens during February 2017–May 2018. We observed a prevalence of sickle cell trait and disease of 20.3% (3492/17 200) and 1.2% (210/17 200), respectively. District-level trait varied from 8.6% (5/58) to 28.1% (77/274). Among confirmed sickle cell disease specimens, we noted 42.7% (61/143) had 1-gene deletion and 14.7% (21/143) had 2-gene deletion α-thalassaemia trait. We documented G6PD A^–^ deficiency in 19.2% (14/73) of males.

**Conclusion:**

Our calculated prevalence is twice as high as previously reported and reinforces the need for enhanced sickle cell diagnostic services. Our district-level data will inform public health policy, allowing screening and disease-modifying hydroxyurea therapy to be focused on high-prevalence areas, until universal newborn screening is available.

## Résumé

**Objectif:**

Déterminer la prévalence de la drépanocytose et du trait drépanocytaire chez les nouveau-nés, ainsi que la prévalence de variants d'hémoglobine et de modificateurs génétiques de la drépanocytose dans les neuf régions du nord-ouest de la République-Unie de Tanzanie.

**Méthodes:**

Nous avons réutilisé des échantillons de gouttes de sang séché provenant d'enfants (âgés de 0 à 24 mois) nés de mères atteintes du virus de l'immunodéficience humaine (VIH), prélevés dans le cadre du programme de diagnostic précoce de l'infection à VIH chez le nourrisson pour détecter une éventuelle drépanocytose. Nous avons procédé à une focalisation isoélectrique pour savoir si les échantillons présentaient une hémoglobine normale, un trait drépanocytaire, une drépanocytose ou un variant d'hémoglobine rare. Ensuite, nous avons envoyé les échantillons identifiés comme porteurs de la maladie ou d'un variant à l'hôpital pour enfants de Cincinnati, aux États-Unis, afin d'analyser l'acide désoxyribonucléique et de mesurer la prévalence d'une α-thalassémie, d'un déficit en glucose-6-phosphate déshydrogénase (G6PD) ou de modificateurs génétiques de l'hémoglobine fœtale.

**Résultats:**

Nous avons étudié 17 200 spécimens au total entre février 2017 et mai 2018. Nous avons observé une prévalence de 20,3% (3492/17 200) pour la drépanocytose, et de 1,2% (210/17 200) pour le trait drépanocytaire. D'un district à l'autre, la présence du trait variait de 8,6% (5/58) à 28,1% (77/274). Nous avons constaté que 42,7% (61/143) des spécimens chez qui une drépanocytose avait été confirmée étaient privés d'un gène, et 14,7% (21/143) de deux gènes du trait α-thalassémique. Enfin, nous avons répertorié un déficit en G6PD A^–^ chez 19,2% (14/73) des mâles.

**Conclusion:**

La prévalence que nous avons calculée est deux fois supérieure aux chiffres mentionnés précédemment, et souligne la nécessité d'instaurer de meilleurs services de diagnostic de la drépanocytose. Nos données réparties par district fourniront des informations en matière de politique de santé publique, afin que le dépistage et le traitement modificateur de la maladie, à base d'hydroxyurée, se concentrent sur les zones de forte prévalence jusqu'à ce qu'un dépistage universel des nouveau-nés soit disponible.

## Resumen

**Objetivo:**

Determinar la prevalencia de la enfermedad y del rasgo de las células falciformes en los recién nacidos a nivel regional y de distrito, y la prevalencia de las variantes de hemoglobina y de los modificadores genéticos de la drepanocitosis en las nueve regiones del noroeste de la República Unida de Tanzania.

**Métodos:**

Se reutilizaron las muestras de las manchas de sangre seca de los niños (de 0 a 24 meses de edad) nacidos de madres que padecían el virus de la inmunodeficiencia humana (VIH); estas muestras se obtuvieron a través del programa de Diagnóstico precoz del VIH en niños para diagnosticar las células falciformes. Se aplicó la técnica del enfoque isoeléctrico para determinar si las muestras tenían hemoglobina, rasgos de células falciformes, drepanocitosis normales o una variante de hemoglobina poco frecuente. Se enviaron muestras diagnosticadas como enfermedad o variante al Hospital Infantil de Cincinnati (Cincinnati Children's Hospital) en los Estados Unidos de América para analizarlas a base de ácido desoxirribonucleico y así determinar la prevalencia de talasemia α, de deficiencia de glucosa-6-fosfato deshidrogenasa (G6PD, por sus siglas en inglés) o de modificadores genéticos de hemoglobina fetal.

**Resultados:**

Se analizaron un total de 17 200 muestras entre febrero de 2017 y mayo de 2018. Se observó una prevalencia del rasgo de las células falciformes y de la drepanocitosis del 20,3 % (3492/17 200) y del 1,2 % (210/17 200), respectivamente. El rasgo a nivel de distrito varió del 8,6 % (5/58) al 28,1 % (77/274). Se observó que en las muestras de drepanocitosis confirmadas, el 42,7 % (61/143) presentaba la eliminación de un gen y el 14,7 % (21/143) la eliminación de dos genes en el rasgo de talasemia α. Se registró una deficiencia de G6PD A- en el 19,2 % (14/73) de los varones.

**Conclusión:**

La prevalencia que se calcula aquí es el doble de la que se notificó anteriormente y refuerza la necesidad de mejorar los servicios para el diagnóstico de la drepanocitosis. Estos datos a nivel de distrito contribuirán a la política de salud pública, ya que permitirán que los cribados y la terapia con hidroxicarbamida que modifica la enfermedad se centren en las zonas de alta prevalencia, hasta que se disponga de un cribado universal de los recién nacidos.

## ملخص

الغرض تحديد معدل انتشار سمات ومرض الخلايا المنجلية لدى حديثي الولادة على مستوى الأقاليم والمناطق، وانتشار متغيرات الهيموجلوبين والمعدلات الجينية لمرض فقر الدم المنجلي، في المناطق التسع الواقعة شمال غرب جمهورية تنزانيا المتحدة.

الطريقة قمنا بإعادة استخدام عينات بقع الدم المجففة من الأطفال (من حديثي الولادة إلى 24 شهرًا) المولودين لأمهات مصابات بفيروس نقص المناعة البشرية (HIV)، والتي تم جمعها كجزء من برنامج التشخيص المبكر لفيروس نقص المناعة البشرية للرضع، لتشخيص مرض الخلايا المنجلية. أجرينا تركيزًا متساوي الجهد الكهربائي لتحديد ما إذا كانت العينات تحتوي على هيموجلوبين طبيعي، أو سمة للخلايا المنجلية، أو مرض الخلايا المنجلية، أو أحد المتغيرات النادرة للهيموجلوبين. كما قمنا بشحن عينات تم تشخيصها على أنها مصابة بالمرض أو أحد المتغيرات، إلى مستشفى سينسيناتي للأطفال في الولايات المتحدة الأمريكية، لإجراء تحليلات باستخدام حمض الديوكسي ريبونيوكليك لتحديد مدى انتشار كل من ثلاسيميا ألفا، أو النقص في نازع هيدروجين الجلوكوز 6 فوسفات (G6PD)، أو المعدلات الوراثية للهيموجلوبين الجنيني.

النتائج قمنا بتحليل إجمالي 17200 عينة خلال الفترة من فبراير/شباط 2017 إلى مايو/أيار 2018. ولاحظنا انتشار سمة ومرض الخلايا المنجلية بنسبة 20.3% (3492/17200)، و1.2% (210/17200)، على الترتيب. تنوعت السمات على مستوى المناطق من 8.6% (5/58) إلى 28.1% (77/274). ولاحظنا من بين عينات مرض الخلايا المنجلية المؤكد، أن 42.7% (61/143) قد عانت من حذف جين واحد، و14.7% (21/143) عانت من حذف جينين من سمة ألفا ثلاسيميا. وكما بتوثيق النقص في G6PD A في 19.2% (14/73) من الذكور.

الاستنتاج إن معدل الانتشار الذي قمنا باحتسابه هو ضعف ما تم الإبلاغ عنه سابقًا، ويعزز الحاجة إلى خدمات محسنة لتشخيص مرض الخلايا المنجلية. ستؤدي البيانات على مستوى المقاطعات لدينا إلى توجيه سياسة الصحة العامة، مما يسمح لكل من الفحص والمعالجة باستخدام هيدروكسي اليوريا المعدل للأمراض، بالتركيز على المناطق ذات الانتشار المرتفع، حتى يتوفر فحص شامل لحديثي الولادة.

## 摘要

**目的:**

确定坦桑尼亚联合共和国西北九个地区，新生儿镰状细胞性状和镰状细胞疾病的患病率，以及血红蛋白变体和镰状细胞疾病遗传修饰因子的患病率。

**方法:**

作为 HIV 早期婴儿诊断计划的一部分，我们将携带人类免疫缺陷病毒 (HIV) 的母亲所生婴儿（0-24 个月）的干血斑样本重新用于诊断镰状细胞。我们对其实施等电聚焦，以确定样本的血红蛋白是否正常、是否具有镰状细胞性状、镰状细胞疾病或罕见的血红蛋白变体。我们将诊断出患有疾病或变异的样本运送到美国辛辛那提儿童医院，进行脱氧核糖核酸分析，以确定α地中海贫血、6-磷酸葡萄糖脱氢酶 (G6PD) 缺失症或胎儿血红蛋白遗传修饰因子的患病率。

**结果:**

我们在 2017 年 2 月至 2018 年 5 月期间共分析了 17200 个样本。我们观察到镰状细胞性状和镰状细胞疾病的患病率分别为 20.3％ (3492/17 200) 和 1.2％ (210/17 200)。区级镰状细胞性状患病率则从 8.6% (5/58) 到 28.1% (77/274) 不等。在确诊的镰状细胞疾病样本中，我们发现 42.7% (61/143) 具有 1 基因缺失型 α 地中海贫血性状，14.7% (21/143) 具有 2 基因缺失型 α 地中海贫血性状。我们注意到 19.2% (14/73) 的男婴患有 G6PD A–缺乏症。

**结论:**

我们计算得出的患病率是先前报道的两倍，这凸显了加强镰状细胞诊断服务的必要性。我们的区级数据将为公共卫生政策提供信息，从而确保在新生儿普查之前，对高发地区集中展开筛查和疾病修饰羟基脲疗法。

## Резюме

**Цель:**

Определить на национальном уровне и на уровне районов распространенность среди новорожденных носительства признака серповидно-клеточной анемии и самого этого заболевания, а также распространенность вариантов гемоглобина и генетических модификаторов серповидно-клеточной анемии в девяти регионах на северо-западе Объединенной Республики Танзания.

**Методы:**

Для диагностики серповидно-клеточной анемии авторы использовали взятые методом высушивания пятна образцы крови детей в возрасте от 0 до 24 месяцев, чьи матери живут с вирусом иммунодефицита человека (ВИЧ); эти образцы были собраны в рамках программы ранней диагностики ВИЧ у детей. Для определения наличия в образцах нормального гемоглобина, носительства признака серповидно-клеточной анемии, собственно серповидно-клеточной анемии или редкого варианта гемоглобина использовался метод изоэлектрической фокусировки. Образцы, которые были диагностированы как соответствующие проявлениям заболевания или как содержащие вариантный гемоглобин, были направлены в Детскую больницу Цинциннати, США, для проведения ДНК-анализа с целью определения распространенности в них α-талассемии, дефицита глюкозо-6-фосфат-дегидрогеназы (Г6ФД) или наличия генетических модификаторов фетального гемоглобина.

**Результаты:**

Авторы проанализировали 17 200 образцов в период с февраля 2017 г. по май 2018 г. Носительство признака серповидно-клеточной анемии было обнаружено в 20,3% случаев (3492 из 17 200 образцов), а серповидно-клеточная анемия — в 1,2% (210 из 17 200 образцов). Наличие признака в разных районах варьировалось от 8,6% (5 из 58 образцов) до 28,1% (77 из 274 образцов). Среди образцов с подтвержденным диагнозом серповидно-клеточной анемии в 42,7% случаев (61 из 143) наблюдался признак α-талассемии с делецией 1 гена и в 14,7% (21 из 143) — с делецией 2 генов. Дефицит Г6ФД был зафиксирован у 19,2% (14 из 73) мальчиков.

**Вывод:**

Рассчитанная авторами распространенность заболевания вдвое превышает опубликованные ранее значения и еще раз указывает на необходимость укрепления и расширения услуг по диагностике СКА. Полученные данные по районам станут основой для выработки политики в области общественного здравоохранения, которая бы позволяла сосредоточить скрининговое обследование и лечение гидроксимочевиной (изменяющее течение заболевания) в областях с высоким распространением заболевания до тех пор, пока не станет возможным всеобщее скрининговое обследование новорожденных.

## Introduction

Sickle cell disease is an inherited disorder of haemoglobin, caused by a mutation in the β-globin subunit of adult haemoglobin. In classic autosomal recessive fashion, inheritance of one abnormal and one normal allele confers sickle cell trait, a carrier state without clinical symptoms. Inheritance of two mutated alleles causes sickle cell disease, characterized by varying amounts of chronic haemolytic anaemia, recurrent debilitating pain and an array of clinical sequelae, including increased risk of infection, stroke, lung disease, splenic dysfunction and bone infarction.^1^

Sickle cell disease imposes a significant global burden of disease that remains underrecognized,^2^ especially in Africa. Approximately 400 000 infants are born each year with sickle cell disease;^3^^–^^5^ 75% of these infants are born in the tropical regions of sub-Saharan Africa,^6^ home to most of the > 25 million people who live with sickle cell disease globally.^7^ Sickle cell disease causes substantial morbidity^8^ and is responsible for 5–16% of mortality in children younger than 5 years.^9^^,^^10^ Cumulative data from prior studies suggest that more than half of the children with sickle cell disease in sub-Saharan Africa die in early childhood,^11^ with substantial differences in mortality between historic rural communities^12^ and modern urban centres.^13^ Early enrolment in a comprehensive care programme that includes preventive care (immunizations and prophylactic antimicrobials) and disease-modifying therapy (hydroxyurea or prophylactic blood transfusions) can reduce symptoms and improve survival.

The World Health Organization acknowledged the global importance of addressing sickle cell disease almost 15 years ago,^10^^,^^14^^,^^15^ and in 2010 African leaders formally proposed sickle cell disease prevention and control strategies for the African Region.^5^ In response, the United Republic of Tanzania has embedded sickle cell disease targets within the national noncommunicable disease policy,^16^ increased advocacy,^17^ created a centre of excellence,^18^ educated health-care workers and increased research output.^19^ The majority of these efforts have been focused in Muhimbili National Hospital in the coastal city of Dar es Salaam, but prevalence estimates suggest that the greatest burden of sickle cell disease is in the north-western regions of the country around Lake Victoria.^6^^,^^20^^,^^21^

The United Republic of Tanzania does not yet have a national newborn screening programme. In the absence of recent reports, Tanzanian estimates of sickle cell disease are based on sparse data from studies performed over the past 65 years in only seven of the 30 regions of the country.^22^^–^^32^ Recognizing that data extracted from isolated reports can poorly reflect variation within a country,^33^^,^^34^ we initiated and conducted the United Republic of Tanzania Sickle Surveillance Study in the north-western regions of the country. Our primary objective was to provide contemporary regional- and district-level data on the newborn prevalence of sickle cell trait and disease to inform the national noncommunicable disease policy goals. Our study used existing public health infrastructure developed as part of the human immunodeficiency virus (HIV) Early Infant Diagnosis programme,^35^ while aiming to build local capacity for the accurate diagnosis of sickle cell disease. Our secondary objectives included characterization of rare haemoglobin variants and the prevalence of co-inherited genetic disorders that may affect sickle cell disease phenotypes and response to hydroxyurea treatment.

## Methods

### HIV Early Infant Diagnosis

In 2006, the International Center for AIDS Care and Treatment Programs, the Tanzanian Ministry of Health and Social Welfare, Bugando Medical Centre and the United States Centers for Disease Control and Prevention implemented the HIV Early Infant Diagnosis programme with the aim of preventing mother-to-child transmission of HIV.^35^ The country operates a decentralized testing structure with four reference laboratories. The Bugando Medical Centre, a zonal referral and teaching hospital located in the city of Mwanza, serves as the reference laboratory for the north-western catchment area, and is also the designated sickle cell centre of excellence for this area. The Bugando catchment area includes a population of approximately 17.6 million people within nine regions, who collectively represent 39.2% (17 623 047/44 928 923) of the country’s total population.^36^

An infant born to a mother living with HIV is brought to a Reproductive and Child Health clinic at 4–6 weeks of age for the first preventive health visit. A dried blood spot is collected from the infant, and transported to a reference laboratory for detection of HIV using a polymerase chain reaction (PCR). The dried blood spots are labelled with key demographic information including date of birth, sex, referring health facility, date of collection, date of dispatch and date of receipt at the laboratory. HIV results are communicated to the referring health facility for prompt initiation of antiretroviral medication. Dried blood spots are then stored at room temperature and made available for additional testing for sickle cell trait and disease. 

### Sickle cell diagnosis 

We analysed all repurposed dried blood spots collected as part of the HIV Early Infant Diagnosis programme during February 2017–May 2018 by using the isoelectric focusing technique.^34^ Our laboratory equipment included Resolve Hemoglobin kits and JB-2 Staining System reagents (both PerkinElmer, Inc., Waltham, United States of America, USA), as well as control specimens and other consumables, all donated to the haematology section of the Bugando laboratory. Staff were trained on-site by a board-certified haematologist, and attended a 2-day seminar in Dar es Salaam, organized by the equipment manufacturer and funded jointly by the manufacturer and the United States Association of Public Health Laboratories. At the seminar, staff acquired theoretical knowledge of the isoelectric focusing technique and had the opportunity to process samples while supervised by an experienced manufacturer representative. The senior Tanzanian paediatrician leading the haematology clinic also completed a further two months of clinical and laboratory training funded and hosted by Cincinnati Children’s Hospital, USA. 

All dried blood spots were analysed with a standard control specimen containing adult, fetal and sickling haemoglobin (haemoglobin A, F and S) and haemoglobin C. Isoelectric focusing results were scored independently by two Bugando Medical Centre staff for the presence and abundance of each type of haemoglobin. The results were interpreted as: normal if haemoglobin A (± haemoglobin F) was present; sickle cell disease if haemoglobin S (± haemoglobin F) was present; sickle cell trait if both haemoglobin A and S (± haemoglobin F) were present; variant if a band was present at any location other than haemoglobin A, S or C (± haemoglobin F); and uninterpretable if poor quality precluded interpretation. Dried blood spot analyses interpreted as sickle cell disease, variant or uninterpretable were repeated for confirmation and frozen for later deoxyribonucleic acid (DNA) studies. Regular teleconferences were convened with collaborators based in the Cincinnati Children’s Hospital, USA, to provide ongoing feedback on the quality of laboratory techniques and interpretation of gels; however, the Tanzanian team was responsible for all final interpretations. 

### DNA-based testing

Any dried blood spot diagnosed as sickle cell disease or variant was stored at −20 °C then shipped to the USA for future DNA testing. Upon arrival, we stored dried blood spots at −80 °C until testing could be performed. We extracted DNA from dried blood spots using an adapted protocol from Instagene (Bio-Rad Laboratories, Hercules, USA).^37^ We performed amplification of β-globin gene exons 1 and 2 using a PCR, and confirmed the presence of a haemoglobin S mutation at rs334 (c.334T > A;p.Glu6Val) using a custom TaqMan PCR probe (Applied Biosystems, Foster City, USA).^37^ We analysed specimens interpreted as uncommon and atypical haemoglobin variants by isoelectric focusing via the previously published algorithm^37^ used to investigate uncommon variants in East Africa. Uncommon variants include the α-chain variants haemoglobin G-Pest (*HBA1*:p.Asp75Asn) and haemoglobin Stanleyville II (*HBA1*:p.Asn79Lys), and the fusion variants haemoglobin P-Nilotic (β-globin gene (*HBB*)–δ-globin gene (*HBD*) fusion: β31-δ50) and haemoglobin Kenya (γ-globin gene (*HBG1*)–*HBB* fusion: ^A^γ81-β86).^37^

We detected α-thalassaemia trait resulting from the 3.7-kilobase α-globin gene deletion and glucose-6-phosphate dehydrogenase (G6PD) A^–^ variant using DNA-based techniques,^37^^–^^39^ and determined the modifiers of baseline haemoglobin F production.^40^ We genotyped the *BCL11A* polymorphisms (rs1427407, rs7557939 and rs11886868) and *HBS1L-MYB* intergenic polymorphisms (HMIP) (rs28384513 and rs9399137) using commercially available real-time PCR assays (Applied Biosystems, Foster City, USA). To identify the XmnI single-nucleotide polymorphism at −158 basepairs to G-γ globin (rs7482144), we amplified the G-γ gene (*HBG2*) using PCR with G-γ-specific forward and reverse primers to ensure that the G-γ rather than an A-γ product was isolated. We then further amplified this product by performing PCR with Classic 1 forward and reverse primers. We genotyped the final product using a custom-made TaqMan PCR probe set (Applied Biosystems, Foster City, USA).

### Data analysis

We entered data into an Excel database (Microsoft, Redmond, USA), treating age as a continuous variable (summarized using median and interquartile range) and treating haemoglobin type, HIV status, sex, region of origin, district of origin, G6PD status and α-thalassaemia status as categorical variables (summarized using frequencies). We calculated the prevalence of sickle cell trait and disease by dividing the number of specimens with sickle cell trait and disease by the total number of non-missing specimens with interpretable results. We determined allelic frequency by dividing the number of times that an allele was observed (once in heterozygotes, twice in homozygotes) by the total number of all alleles (twice the total number of specimens). We compared the proportions of participants living with HIV with and without sickle cell disease using a *χ^2^* test.

### Ethical considerations

Our study protocol was approved with a waiver for informed consent by the joint Catholic University of Health and Allied Sciences–Bugando Medical Centre Research and Ethics Committee, as well as the Tanzanian National Institute for Medical Research, to perform disease surveillance on de-identified archived dried blood spots previously collected by the HIV Early Infant Diagnosis programme. The study was also approved by the Cincinnati Children’s Hospital Medical Center Institutional Review Board. A formal material transfer agreement was obtained so that specimens could be shipped to the USA for genetic analysis.

## Results

### Sample collection and testing 

Our local Bugando Medical Centre staff completed a total of 232 isoelectric focusing gels during February 2017–May 2018. After samples from children older than 24 months were excluded to obtain a more accurate prevalence for infants, the median age of infants included in the sampling population was 52 days (interquartile range, 41–93 days). Staff scored a total of 17 274 unique dried blood spot specimens from the catchment area. The quality of laboratory testing was extremely high with only 20 specimens scored as uninterpretable and 54 with missing results, meaning that we performed our primary analysis on 17 200 specimens.

### Prevalence

**Fig. 1:**
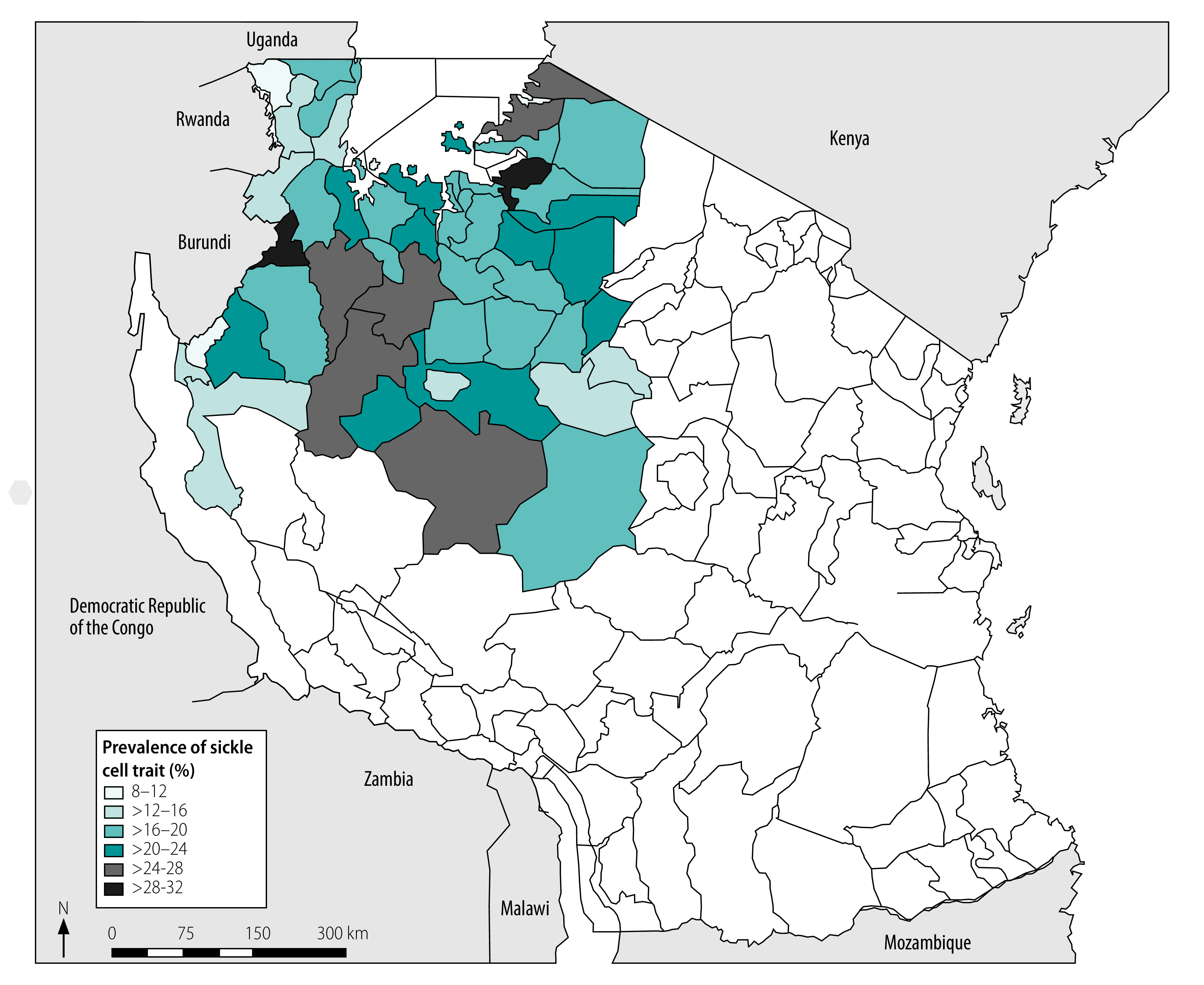
District-level prevalence of sickle cell trait in infants, north-western United Republic of Tanzania, February 2017–May 2018

Note: We only included districts that provided a minimum of 49 specimens.

We observed an overall prevalence of sickle cell trait and disease in our cohort of 20.3% (3492/17 200) and 1.2% (210/17 200), respectively, with a 0.1% (17/17 200) prevalence of atypical or uncommon haemoglobin variants. Our data yielded an allelic frequency of 0.114 ([3492+(2 × 210)]/(2 × 17 200)) for the sickle gene (haemoglobin S) mutation, and demonstrate perfect Hardy–Weinberg equilibrium, as expected in a stable population.^41^ We did not identify any haemoglobin C or other common β-globin variants. Our geospatial mapping revealed mild variation between regions, with the prevalence of sickle cell trait and disease ranging from 16.6% (146/880) to 22.5% (253/1126) and 0.5% (4/880) to 1.5% (17/1126), respectively (Table 1). Analysis of individual districts that provided more than  48 specimens (i.e. excluding Buhigwe, from which only 12 samples were provided) revealed a wider geographic variability, with sickle cell trait and disease ranging from 8.6% (5/58) to 28.1% (77/274) and from zero to 4.3% (9/208), respectively (Fig. 1 and Table 1).

**Table 1:** Regional and district prevalence of haemoglobin types in infants identified, north-western United Republic of Tanzania, February 2017–May 2018

Region or district	Total	No. (%)			
		Normal	Sickle cell trait	Sickle cell disease	Variant
**Region **					
Geita	2 436	1 918 (78.7)	495 (20.3)	23 (0.9)	0 (0.00)
Kagera	880	729 (82.8)	146 (16.6)	4 (0.5)	1 (0.1)
Kigoma	683	550 (80.5)	123 (18.0)	10 (1.5)	0 (0.00)
Mara	1 837	1 422 (77.4)	388 (21.1)	23 (1.3)	4 (0.2)
Mwanza	3 847	3 013 (78.3)	778 (20.2)	51 (1.3)	5 (0.1)
Shinyanga	2 800	2 158 (77.1)	598 (21.4)	41 (1.5)	3 (0.1)
Simiyu	1 126	855 (75.9)	253 (22.5)	17 (1.5)	1 (0.1)
Singida	1 141	906 (79.4)	226 (19.8)	9 (0.8)	0 (0.00)
Tabora	2 450	1 930 (78.8)	485 (19.8)	32 (1.3)	3 (0.1)
**Total**	**17 200**	**13 481 (78.4)**	**3492 (20.3)**	**210 (1.2)**	**17 (0.1)**
**District**					
Bariadi	261	206 (78.9)	52 (19.9)	2 (0.8)	1 (0.4)
Biharamulo	135	111 (82.2)	24 (17.8)	0 (0.0)	0 (0.0)
Buhigwe^a^	12	11 (91.7)	1 (8.3)	0 (0.0)	0 (0.0)
Bukoba	174	142 (81.6)	30 (17.2)	1 (0.6)	1 (0.6)
Bukombe	394	294 (74.6)	97 (24.6)	3 (0.8)	0 (0.0)
Bunda	378	301 (79.6)	73 (19.3)	3 (0.8)	1 (0.3)
Busega	274	195 (71.2)	77 (28.1)	2 (0.7)	0 (0.0)
Butiama	208	161 (77.4)	38 (18.3)	9 (4.3)	0 (0.0)
Chato	514	399 (77.6)	108 (21.0)	7 (1.4)	0 (0.0)
Geita	1 156	929 (80.4)	219 (18.9)	8 (0.7)	0 (0.0)
Igunga	539	426 (79.0)	107 (19.9)	4 (0.7)	2 (0.4)
Ikungi	135	110 (81.5)	23 (17.0)	2 (1.5)	0 (0.0)
Ilemela	513	394 (76.8)	110 (21.4)	9 (1.8)	0 (0.0)
Iramba	291	230 (79.0)	60 (20.6)	1 (0.3)	0(0.0)
Itilima	152	120 (78.9)	29 (19.1)	3 (2.0)	0 (0.0)
Kahama	1 683	1281 (76.1)	374 (22.2)	25 (1.5)	3 (0.2)
Kakonko	49	39 (79.6)	10 (20.4)	0 (0.0)	0 (0.0)
Kaliua	374	284 (75.9)	83 (22.2)	7 (1.9)	0 (0.0)
Karagwe	95	81 (85.3)	14 (14.7)	0 (0.0)	0 (0.0)
Kasulu	159	120 (75.5)	34 (21.4)	5 (3.1)	0 (0.0)
Kibondo	162	129 (79.6)	31 (19.1)	2 (1.2)	0 (0.0)
Kigoma	207	171 (82.6)	34 (16.4)	2 (1.0)	0 (0.0)
Kishapu	424	333 (78.5)	87 (20.5)	4 (0.9)	0 (0.0)
Kwimba	282	230 (81.6)	50 (17.7)	2 (0.7)	0 (0.0)
Kyerwa	58	53 (91.4)	5 (8.6)	0 (0.0)	0 (0.0)
Magu	664	541 (81.5)	116 (17.5)	7 (1.1)	0 (0.0)
Manyoni	285	223 (78.2)	60 (21.1)	2 (0.7)	0 (0.0)
Maswa	216	167 (77.3)	45 (20.8)	4 (1.9)	0 (0.0)
Mbogwe	273	215 (78.8)	53 (19.4)	5 (1.8)	0 (0.0)
Meatu	223	167 (74.9)	50 (22.4)	6 (2.7)	0 (0.0)
Missenyi	163	134 (82.2)	29 (17.8)	0 (0.0)	0 (0.0)
Misungwi	429	334 (77.9)	86 (20.0)	6 (1.4)	3 (0.7)
Mkalama	100	76 (76.0)	23 (23.0)	1 (1.0)	0 (0.0)
Muleba	191	155 (81.2)	34 (17.8)	2 (1.0)	0 (0.0)
Musoma	461	352 (76.4)	103 (22.3)	4 (0.9)	2 (0.4)
Ngara	64	53 (82.8)	10 (15.6)	1 (1.6)	0 (0.0)
Nyamagana	1 107	876 (79.1)	216 (19.5)	13 (1.2)	2 (0.2)
Nyang’hwale	99	81 (81.8)	18 (18.2)	0 (0.0)	0 (0.0)
Nzega	459	366 (79.7)	88 (19.2)	5 (1.1)	0 (0.0)
Rorya	486	371 (76.3)	108 (22.2)	6 (1.2)	1 (0.2)
Sengerema	705	525 (74.5)	169 (24.0)	11 (1.6)	0 (0.0)
Serengeti	89	73 (82.0)	15 (16.9)	1 (1.1)	0 (0.0)
Shinyanga	692	543 (78.5)	137 (19.8)	12 (1.7)	0 (0.0)
Sikonge	233	182 (78.1)	50 (21.5)	0 (0.0)	1 (0.4)
Singida	330	267 (80.9)	60 (18.2)	3 (0.9)	0 (0.0)
Tabora	347	288 (83.0)	53 (15.3)	6 (1.7)	0 (0.0)
Tarime	215	164 (76.3)	51 (23.7)	0 (0.0)	0 (0.0)
Ukerewe	147	113 (76.9)	31 (21.1)	3 (2.0)	0 (0.0)
Urambo	211	165 (78.2)	41 (19.4)	5 (2.4)	0 (0.0)
Uvinza	94	80 (85.1)	13 (13.8)	1 (1.1)	0 (0.0)
Uyui	288	220 (76.4)	63 (21.9)	5 (1.7)	0 (0.0)
**Total**	**17 200**	**13 481 (78.4)**	**3492 (20.3)**	**210 (1.2)**	**17 (0.1)**

^a^ We considered the number of samples returned from Buhigwe (12) to be too small to be statistically representative; all other districts returned ≥ 49 samples.

Note: We used isoelectric focusing to detect different haemoglobin types in infants aged 0–24 months.

Using our regional prevalence estimates of sickle cell trait and disease, and regional census data from the 2012 Population and Housing Census for the United Republic of Tanzania,^36^ we calculated the estimated number of annual births with sickle cell trait or disease in each region within the study area (Table 2). We estimated that the number of births with sickle cell disease per year was 10 056 within the nine regions comprising the study area. Contributions varied by region according to their population size and sickle cell disease prevalence. We projected the lowest number of births with sickle cell disease per year for the region of Singida (526 births), and the highest for the region of Mwanza (1730 births; Table 2).

**Table 2:** Estimated annual region-specific numbers of infants with sickle cell trait and disease, north-western United Republic of Tanzania, February 2017–May 2018

Region	Population^a^	Crude birth rate per 1 000 population^a^	Estimated annual no. of births with sickle cell trait^b^	Estimated annual no. of births with sickle cell disease^b^
Geita	1 739 530	56	19 775	877
Kagera	2 458 023	44	17 953	541
Kigoma	2 127 930	48	18 385	1 532
Mara	1 743 830	49	18 029	1 111
Mwanza	2 772 509	48	26 882	1 730
Shinyanga	1 534 808	44	14 452	1 013
Simiyu	1 584 157	52	18 535	1 236
Singida	1 370 637	48	13 027	526
Tabora	2 291 623	50	22 687	1 490
**Total**	**–**	**–**	**169 725**	**10 056**

^a^ From 2012 Population and Housing Census for the United Republic of Tanzania.^36^

^b^ Estimated from population, crude birth rate and prevalence as calculated in Table 1.

### Relation to HIV status

For the 16 479 samples for which HIV results were available, we analysed the co-morbidity of HIV and sickle cell disease to compare the potential effect of HIV status on mortality, as previously performed in Uganda.^34^ The prevalence of sickle cell disease was 1.2% among both HIV-infected (9/732) and HIV-negative (192/15 747) infants, indicating that HIV status has no effect on early mortality (Table 3).

**Table 3:** HIV-specific prevalence of haemoglobin types in infants, north-western United Republic of Tanzania, February 2017–May 2018

HIV status	Total	No. (%)		
		Normal	Sickle cell trait	Sickle cell disease
Negative	15 747	12 388 (78.7)	3167 (20.1)	192 (1.2)
Positive	732	563 (76.9)	160 (21.9)	9 (1.2)
**Total**	**16 479^a^**	**12 951 (78.6)**	**3327 (20.2)**	**201 (1.2)**

HIV: human immunodeficiency virus.

^a^ Only specimens for which HIV test results were available are included.

Note: We used isoelectric focusing to detect different haemoglobin types in infants aged 0–24 months.

### DNA-based analysis

Of the 210 specimens that were interpreted as sickle cell disease by isoelectric focusing, 143 were genotype-confirmed and made available for further DNA-based testing. Uncommon or atypical haemoglobin variants were rare (0.1%; 17/17 200) and included four haemoglobin G-Pest (*HBA1*:p.Asp75Asn), two haemoglobin Kenya (*HBG1–HBB* fusion ^A^γ81-β86) and a haemoglobin P-Nilotic (*HBB–HBD* fusion β31-δ50). We identified 1-gene deletion α-thalassaemia trait in 42.7% (61/143) and 2-gene deletion α-thalassaemia trait in 14.7% (21/143). We detected G6PD A^–^ deficiency in 19.2% (14/73) of males, and 25.7% (18/70) of females were heterozygous carriers (Table 4).

**Table 4:** Prevalence of α-thalassaemia and G6PD deficiency observed in infants, north-western United Republic of Tanzania, February 2017–May 2018

Genotype	Clinical effect	No. (%)
**α-thalassaemia (*n* = 143)**		
5 copies, αα/ααα	1-gene duplication, unaffected	0 (0.0)
4 copies, αα/αα	0-gene deletion, unaffected	61 (42.7)
3 copies, αα/–α^3.7^	1-gene deletion, α-thalassaemia minima	61 (42.7)
2 copies, –α^3.7^/–α^3.7^	2-gene deletion, α-thalassaemia trait	21 (14.7)
**G6PD deficiency (*n* = 143)**		
Males (*n* = 73)		
B	Wild type, unaffected	52 (71.2)
A^+^	A^+^ variant, unaffected	7 (9.6)
A^–^	A^–^ variant, affected	14 (19.2)
Females (*n* = 70)		
BB	Homozygous, wild type, unaffected	33 (47.1)
BA^+^	Heterozygous, wild type/A^+^ variant, unaffected	16 (22.9)
A^+^A^+^	Homozygous, A^+^ variant, unaffected	3 (4.3)
BA^–^	Heterozygous, wild type/A^–^ variant, carrier	15 (21.4)
A^+^A^–^	Heterozygous, A^+^ variant/A^–^ variant, carrier	3 (4.3)
A^–^A^–^	Homozygous, A^–^ variant, affected	0 (0.0)

G6PD: glucose-6-phosphate dehydrogenase.

Note: We analysed dried blood spots from infants aged 0–24 months.

We provide the minor allelic frequencies for genetic modifiers that affect haemoglobin F production for the 143 samples available for genetic testing in Table 5. Three single-nucleotide polymorphisms in *BCL11A* had minor allelic frequencies ranging from 0.266 (rs1427407) to 0.325 (rs4671393). Our frequencies were slightly higher than those observed within the African subgroup of the 1000 Genomes Project,^42^ which reported values of 0.238 (rs1427407) and 0.269 (rs4671393). The frequency of two single-nucleotide polymorphisms within HMIP was more variable; the value at rs28384513 (in the HMIP-1 region) was 0.238, which is slightly higher than the 1000 Genomes Project African subgroup (0.184), while the value at rs9399137 (in the HMIP-2 region) was low (0.045) and almost identical to the 1000 Genomes Project African subgroup (0.042). Finally, the A variant of rs7482144 in *HBG2* was not detected, as expected in an East African cohort.

**Table 5:** Prevalence of haemoglobin modifiers observed in infants, north-western United Republic of Tanzania, February 2017–May 2018

Chromosome	Gene	Single-nucleotide polymorphism	Allele change	Higher fetal haemoglobin allele frequency
2	*BCL11A*	rs1188686	T → C	0.322 = C
2	*BCL11A*	rs1427407	G → T	0.266 = T
2	*BCL11A*	rs4671393	G → A	0.325 = A
6	*HBS1L-MYB*	rs28384513	T → G	0.238 = G
6	*HBS1L-MYB*	rs9399137	T → C	0.045 = C
11	*HBG2*	rs7482144	G → A	0.000 = A

## Discussion

We estimate that the annual number of live births with sickle cell disease is at least twice that previously thought to occur in the United Republic of Tanzania; we project that over 10 000 births among just 40% of the Tanzanian population are affected annually, compared with previous estimates of 8655^6^ and 11 022^9^ annually affected births for the whole country. Our calculated prevalence of sickle cell trait and disease concurs with a recent pilot screening project of 919 infants conducted in Mwanza in 2014, which reported a prevalence of 1.4% (13/919) and 19.7% (181/919), respectively.^20^ Although we observed geographical differences between districts, our variations were not as high as those reported from neighbouring Uganda^34^ where specimens were collected from a roughly equivalent land mass and population. Our high prevalence and relative lack of variation between districts in the north-western part of the country may reflect the high selection pressure from malaria in a holoendemic area, and possibly lower migration rates.

Our data reinforce the urgent need to enhance sickle cell diagnostic services, the obligatory first step in the cascade of care for this neglected patient population. The sickle cell clinic at Bugando Medical Centre has approximately 600 patients currently enrolled in care, some of whom travel from neighbouring regions. However, we estimate that 1730 infants are born with sickle cell disease in Mwanza region alone each year, which indicates that many affected children in sub-Saharan Africa have not even been diagnosed with the condition. The nine regions included in this study have the highest childhood mortality in the country, ranging from 30 to 38 deaths per 1000 live births,^43^ and sickle cell disease contributes significantly to mortality in this age group.^11^^–^^13^

We also investigated the extent to which additional erythrocyte disorders are commonly co-inherited with sickle cell disease; cross-sectional analyses in the United Republic of Tanzania have reported on the impact of such disorders on sickle cell patients.^44^ Ongoing prospective cohort studies will help to clarify the unique interactions between specific sickle cell disease morbidities and co-inherited erythrocyte disorders, endemic arboviral and plasmodial infections, and environmental factors. Expression of haemoglobin F is another well-recognized factor affecting disease severity. Some modifiers of haemoglobin F are similar between Tanzanian and British patients,^45^ but genetic studies among Tanzanians have also identified unique candidate variants and pathways.^46^ Understanding these genetic factors and their influence on disease outcomes will become more important, especially as hydroxyurea therapy is introduced to the region.^47^

An important strength of our study is that we developed a sickle cell disease diagnostic service using existing infrastructure used for HIV infant diagnosis, demonstrating a potential of using this approach across sub-Saharan Africa. For example, in Uganda decision-makers have successfully expanded the use of their HIV infant diagnosis screening platform for sickle cell disease diagnosis, and have launched focused screening in 18 high-prevalence areas.^48^ Until the emergence of government-sponsored universal screening, local providers can implement their own strategies for optimal screening. Regardless of the location (e.g. hospitals, schools, or reproductive and child health clinics), population (e.g. all patients, mothers, newborns or children) or testing modality (isoelectric focusing, electrophoresis, point-of-care testing or high-performance liquid chromatography) used for initial screening, the HIV infant diagnosis infrastructure can be used to transport dried blood spots to a central laboratory equipped for confirmatory testing and diagnosis of sickle cell disease. Expanding testing in this way would represent a crucial step towards achieving some of the targets of the sustainable development goal 3, which are to end preventable deaths among children and reduce premature mortality from noncommunicable diseases.^5^^,^^49^

The testing of infants was another strength of our study. By excluding samples from children older than 24 months from our analyses, we avoided the errors of previous reports^22^^–^^32^ that used adults and children to develop inaccurately low estimates, or that extrapolated sparse results over large geographic areas.

Our study had two limitations. First, we analysed dried blood spots only after they had been processed by the HIV Early Infant Diagnosis laboratory. In some cases, several months had elapsed before the dried blood spots became available for sickle cell disease testing. During that time, dried blood spots were stored at suboptimal conditions for haemoglobin preservation (usually room temperature); however, our isoelectric focusing testing was robust and able to score 99.6% (17 200/17 274) of all dried blood spot specimens. Second, our study population was restricted to children born to mothers living with HIV; however, it has been reported that HIV status is unlikely to affect the genetic inheritance of the sickle allele.^34^

Since universal newborn screening is not immediately feasible in most countries, we recommend initially focusing screening efforts on high-prevalence districts to invest in those communities most affected by sickle cell disease, using existing public health infrastructure with minimal start-up cost and training. The cost–effectiveness of implementing a screening programme in conjunction with treatment for children who are subsequently diagnosed with sickle cell disease varies between regions and countries, especially regarding salary for personnel, but a detailed analysis in Angola provides some helpful comparisons.^50^

Inaccurate national and subnational estimates obscure the true burden of sickle cell disease, which is a common and important cause of death in young children in low-income countries. It is imperative that health ministries and international groups such as the Global Burden of Disease project^51^ receive high-quality district-level data to guide global health priorities and public health policy; our data will inform such strategies. 
